# Controlled release fertilizer delivery system derived from rice straw cellulose nanofibres: a circular economy based solution for sustainable development

**DOI:** 10.1080/21655979.2023.2242124

**Published:** 2023-08-07

**Authors:** Neha Sharma, Benjamin James Allardyce, Rangam Rajkhowa, Ruchi Agrawal

**Affiliations:** a TERI Deakin Nanobiotechnology Centre, Sustainable Agriculture Division, Gurugram, Haryana, India; b Institute for Frontier Materials, Deakin University, Geelong, Victoria, Australia

**Keywords:** Rice straw, cellulose nanofibres, Fourier transform infrared spectroscopy, scanning electron microscopy, Brunauer–Emmett–Teller analysis, loading and release kinetics

## Abstract

Recently, the development of sustainable and environmentally friendly biomaterials has gained the attention of researchers as potential alternatives to petroleum-based materials. Biomaterials are a promising candidate to mitigate sustainability issues due to their renewability, biodegradability, and cost-effectiveness. Thus, the purpose of this study is to explore a cost-effective biomaterial-based delivery system for delivering fertilizers to plants. To achieve this, rice straw (agro-waste) was selected as a raw material for the extraction of cellulose. The cellulose was extracted through alkali treatment (12% NaOH), followed by TEMPO-based oxidation. The cellulose nanofibers were characterized using Fourier transform infrared (FTIR) spectroscopy, scanning electron microscopy, and transmission electron microscopy. In scanning electron microscopy, a loosening of the fibrillar structure in cellulose nanofibers (CNFs) was observed with a diameter of 17 ± 4 nm. The CNFs were loaded with nitrogen-based fertilizer (ammonium chloride) in 1:1, 1:2, and 2:1 (w/w) proportions. The loading was estimated through surface charge variation; in the case of the 1:1 sample, maximum reductions in surface charge were seen from −42.0 mV to −12.8 mV due to the binding of positive ammonium ions. In the release kinetics study, a controlled release pattern was observed at 1:1, which showed a 58% cumulative release of ammonium ions within 8 days. Thus, the study paves the way for value-added uses of rice straw as an alternative to the current environmentally harmful practices.

## Highlights

- Bio-based cellulose nanofibers (CNFs) from rice straw via circular economy approach.
- Controlled release fertilizers for sustainable agriculture.
- Nanotechnology for precision agriculture and decarbonization via agricultural waste management.

## Graphical abstract

**Figure:**
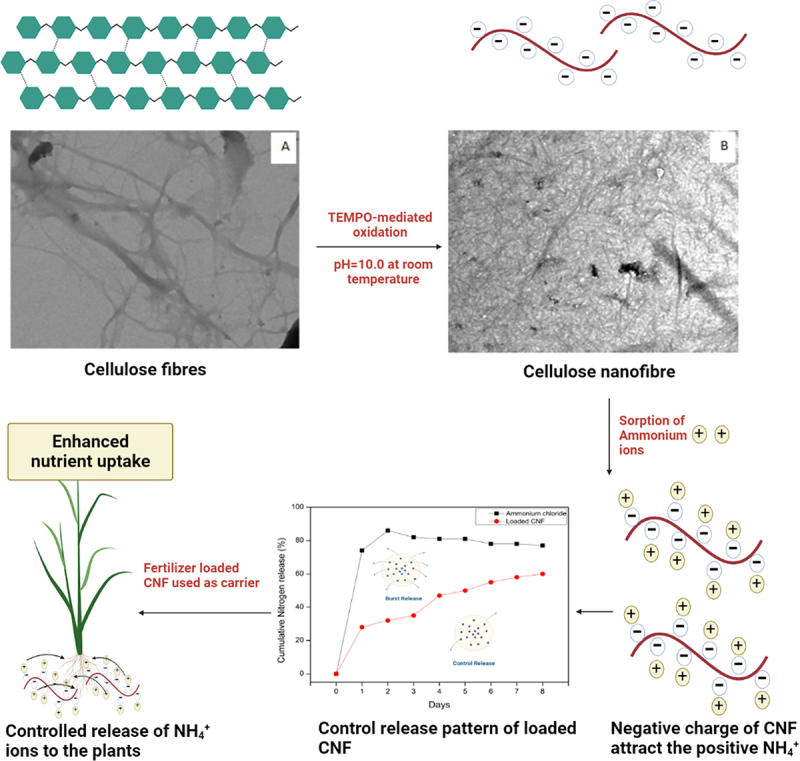

## Introduction

In the 21st century, agriculture has enormous challenges as the global population is rapidly expanding and is expected to reach over 9 billion people by 2050 [1,2]. Due to limited area, poor soil quality, and unavailability of water, the agricultural land area is not increasing proportionately with the increasing population but instead is diminishing. Farmers attempted to address the issue of food availability and enhance the yields per hectare in the short term by increasing the fertilizer application to the fields [3,4]. Despite the fact that our atmosphere, including the soil comprises more than 80% nitrogen, a major nutrient source required by plants for growth, the majority of that proportion is unusable by plants due to its non-reactive nature, and therefore, it needs to be supplemented on a regular basis through the addition of fertilizers. However, plants can only utilize 30–35% of the total nitrogen available in the fertilizer due to their low nutrient uptake efficiency, and hence the substantial energy input is lost [3,5,6]. The major part of the unused nitrogen is swiftly digested by the microbes (bacteria, fungi, and protozoa) or lost to physical and chemical processes such as leaching and volatilization [1]. The loss of nitrogen through leaching causes the contamination of subsurface water bodies and poses a risk to aquatic life [5]. Henceforth, in the longer run, this overuse of fertilizers has resulted in significant reductions in soil quality and substantial environmental issues. To eliminate or reduce this leaking and to enhance the nutrient uptake efficiency of the plant, a polymer-based carrier material (cellulose and cellulose nanofibers) could be introduced that would serve as a controlled release system

Cellulose nanofibers can be extracted through various mechanical or chemical approaches, viz. homogenization, ultrafine grinding or refining, microfluidization, ultrasonic, acid hydrolysis, 2,2,6,6,tetramethylpiperidine-1-oxyl (TEMPO)-mediated oxidation, etc. [7]. TEMPO-mediated oxidation has become increasingly popular in recent years for the development of cellulose nanofibers. The TEMPO process oxidizes the C6 of the D-glucose unit by replacing the OH group with a COOH group. The reaction employs sodium hypochlorite as an oxidizer in the presence of NaBr under alkaline conditions (pH 10 ± 2) [8]. Cellulose nanofibers (CNFs) are promising biomaterials due to their extraordinary properties, such as biodegradability, low density, strong tensile strength, stiffness, biocompatibility, and high availability [8]. Furthermore, the enormous availability of cellulose in sustainable raw agricultural waste, as well as the possibilities of the chemical modification of their structure, allows for the exploitation of cellulose and CNF for various applications [8]. CNF serves as a barrier that holds the nutrients through ionic interaction that further slows and controls fertilizer release [2].

Cellulose nanofibers (CNFs) will be used because of their unique properties, such as large aspect ratios, great specific surface area, excellent biodegradability, mechanical properties, eco-friendliness, non-toxicity, and light weight. However, cellulose has weak water dispersion properties that limit its application, so to overcome this drawback, the cellulose fibers are converted into nanocellulose, along with surface modification and molecular grafting, which have been used to address the disadvantage [9].

Li et al. [10] produced a biodegradable film for urea coating using ethyl cellulose (EC) grafted with vinyl acetate and butyl acrylate. With increasing EC content (EC concentration less than 5%), the hydrophilicity of the EC membrane reduced, resulting in a low urea permeable rate. Qiao et al. [11] used EC as an inner coating for controlled release fertilizer due to its water-insolubility. A report by Zhang et al. [12] developed co-crosslinked carboxymethyl cellulose film with polyvinyl alcohol for ammonium salt release. The rate of ammonium salt penetration reduced as crosslinker concentration increased.

**Figure 1.**
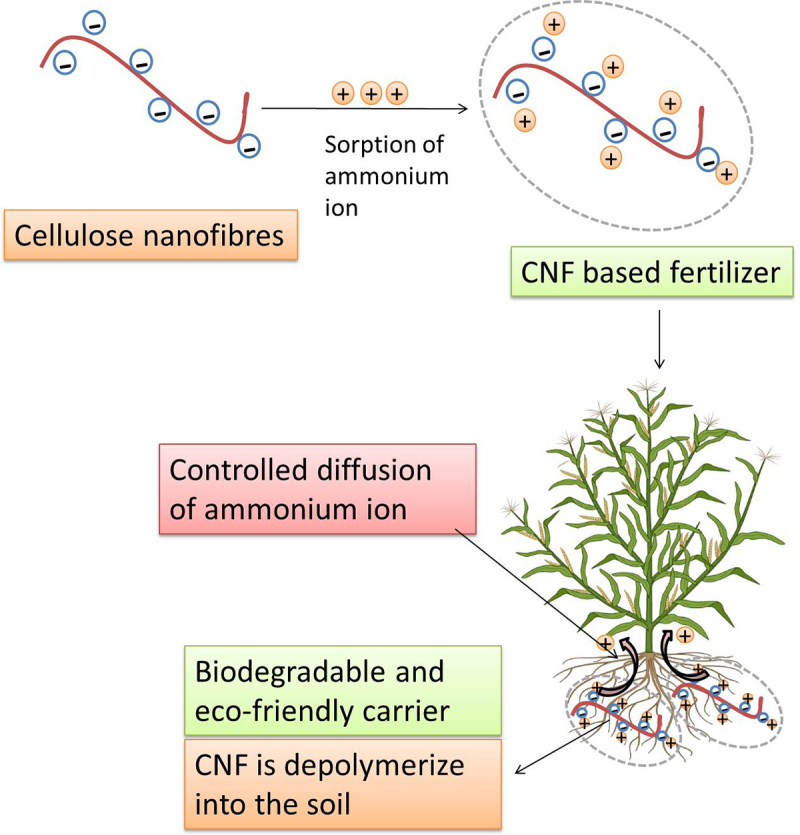
Depicting mechanism of controlled-release mechanism of CNF-based fertilizer.

In this study, a CNF-based carrier system will be used as a controlled release system for fertilizer which overcomes the low nutrient uptake efficiency of conventional fertilizers (Figure 1). The goal of the research is to develop a controlled-release fertilizer using rice straw-derived cellulose nanofibres with NH_4_Cl as the primary nitrogen source. Additionally, this study investigates the morphological (pore diameter and surface area) and, functional, parameters of the cellulose nanofibers (CNFs) produced using the TEMPO oxidation process. Finally, the loading and release efficiency of ammonium chloride as a controlled release fertilizer on CNF was investigated [2]. The novelty of this study is that despite their high flexibility in chemical modification and high surface area, CNFs have not yet been explored as a carrier system for the smart delivery of fertilizers to plants.

## Materials and methods

### Chemicals

Rice straw (Oryza sativa) was collected from the fields of Mathura District (latitude of 27.49 ºN, and longitude 77.67 ºE), Uttar Pradesh, India. Further, it was air-dried and shredded to the size of 5 mm with a knife mill and stored in an airtight sterilized container for future investigations. Sodium hydroxide, sodium chlorite, potassium hydroxide, potassium iodide, and mercuric chloride were purchased from Qualigens. 2,2,6,6-tetramethylpiperidine-1-oxoammonium ion (TEMPO) was purchased from Sigma-Aldrich. Sodium bromide was purchased from Thomas Baker with the purity of 96%. Ammonium chloride was extra pure and purchased from Hi-media. All chemicals used were analytical grade.

### Cellulose extraction from rice straw

Cellulose was isolated from rice straw by an alkali treatment process. Rice straw was treated with 12% NaOH at 10% (w/w) total solids in a high pressure reactor at 121°C for 1 h. The slurry was then neutralized with water before drying overnight at 50°C. The dried residue was bleached using a 5% solution of sodium chlorite at 80°C for 10 min. The white residue was recovered, washed, and dried. This white colored powder was cellulose, which was stored in a sterile and tight container for further investigation [13].

### TEMPO mediated oxidation of cellulose

TEMPO (2, 2, 6, 6-tetramethylpiperidine-1-oxoammonium ion)-mediated oxidation was performed as described by Jiang et al. with slight modifications. Around 500 mg of extracted cellulose was added to 37.5 mL of water and stirred for 5 min using a magnetic stirrer (Tarson digital spinot). Around 6.25 mg of TEMPO and 62.5 mg of sodium bromide were then added and stirred continuously for another 5 min. Further, a 5 mM solution of sodium hypochlorite was added dropwise to initiate the oxidation process. The pH was then reduced to 10 ± 0.2 with 0.5 M NaOH. The oxidation reaction was completed when the pH stabilized, and the reaction was then neutralized by the addition of 0.5 M HCl. The suspension was centrifuged at 8000 rpm and washed several times. The recovered cellulose fibers were further sonicated for defibrillation and freeze-dried for percentage yield of the process [14]. The yield percentage of the overall cellulose nanofiber extraction process was calculated using the following formula:

(1) $$Yield\left(\%\right)=Final\;weight\;of\;cellulose\;fibres\;after\;TEMPO\;treatment\;÷\;Initial\;weight\;of\;cellulose\;fibres\times 100$$

### Mechanical defibrillation of TEMPO treated cellulose through sonication

**Figure 2.**
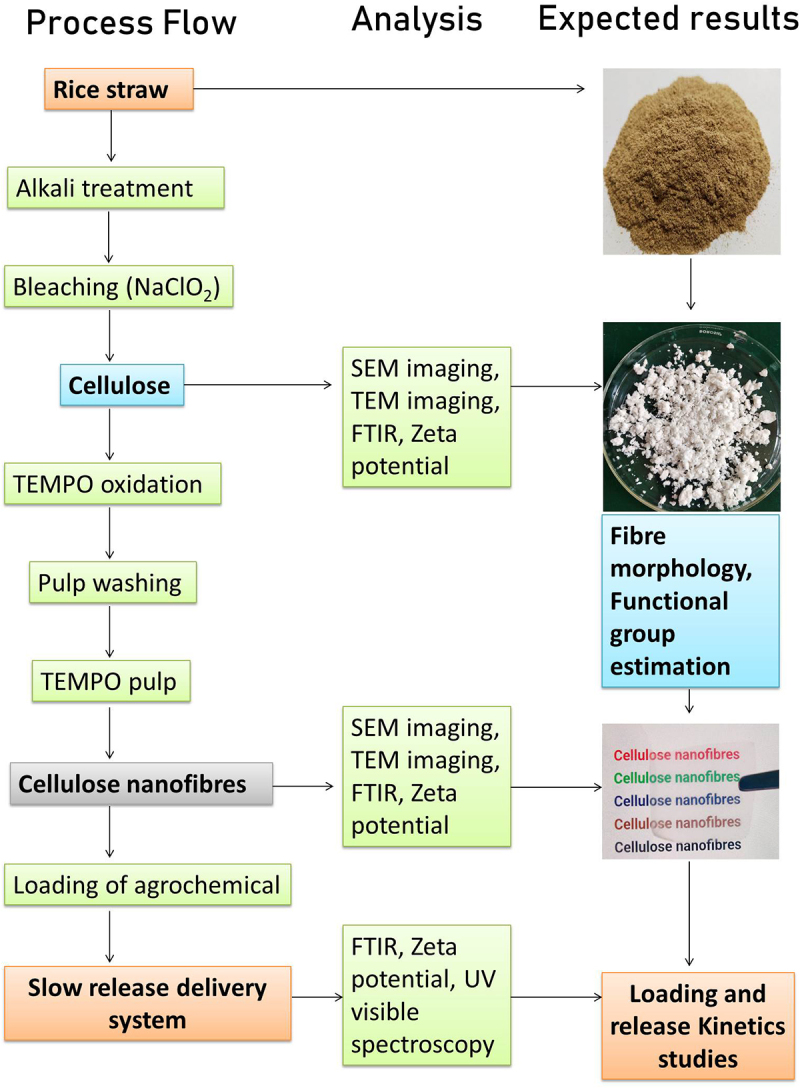
Schematic representation of the process flow and expected results.

0.1% solution of TEMPO oxidized cellulose fibers was prepared and sonicated with a 6 mm probe sonicator (Model VC505, Sonics & Materials, Inc.) with a power output of 500 W and a frequency of 20 kHz for 20 min. To avoid heating during the process, the reaction was placed in an ice bath. A schematic representation of the experimental flow is shown in Figure 2.

### Estimation of nitrogen percentage by Kjeldahl method

The quantitative estimation of nitrogen was performed using Kjeldahl method where 1.0 g of sample was diluted with 50 ml of deionized water. Followed by, addition of 2 g of catalyst (copper sulfate and potassium sulfate) and 20 ml of sulfuric acid into Kjeldahl tube. The blank sample was prepared using water and digested at 390°C for 2 h. The tubes were allowed to cool down and further diluted with deionized water and known amount of sodium hydroxide was added. Subsequently, ammonia was collected using 2% of boric acid and titrated against 0.1 M hydrochloric acid [15].

### Absorption and release investigation of ammonium chloride onto CNF

Absorption tests were performed using varied concentrations of ammonium chloride. Ammonium chloride and CNF (1:1, 1:2, and 2:1 w/w) were suspended in 10 mL solutions for 24 h at room temperature (25°C) using a magnetic stirrer at 300 rpm. The pellets collected by centrifugation at 10,000 rpm were freeze-dried and characterized through FTIR spectroscopy and zeta analysis [16].

An *in-vitro* release of ammonium chloride loaded onto CNF was measured using the dialysis method [17]. 2.00 mL of loaded CNF were immersed in a closed vessel containing 198 mL of milli-Q water at room temperature (25°C) under constant stirring at 250 rpm. At regular time intervals (0, 1, 2, 3 till 8 days), aliquots (1.00 mL) were withdrawn from the vessel and replaced with fresh milli-Q (1.00 mL) to maintain a constant volume. The concentration of ammonium ions released in milli-Q water was determined by Kjeldahl method as described above [18]. The study was done with respect to the control, where 2.00 mL ammonium chloride solution was kept in the dialysis bag. The release experiments were conducted in triplicate.

### Characterization of cellulose nanofibres

#### Fourier transform infrared spectroscopy (FTIR)

The cellulose and CNF were characterized through Fourier Transform Infrared Spectroscopy (Nicolet, 6700) with a scanning wavenumber from 4000 to 400 cm^−1^ for identification of functional groups. The resolution was set at 4 cm^−1^ with 128 scans per spectrum [19].

### Zeta potential measurement

The zeta potential of cellulose and CNF (0.1 wt% aqueous solution) was measured using a Zetasizer Nano ZEN 3690 (Malvern Panalytical, UK). Three measurements were performed for each solution to determine the surface charge of the cellulose and CNFs [20].

### Scanning electron microscopy (SEM)

The cellulose and CNF were analyzed by SEM after drop-casting on a metal stub covered with aluminum tape. The samples were dried using pressurized air, sputter-coated with a 12–15 nm gold layer using a mini sputter coater (SC7620, Quorum Technologies) under vacuum, and examined under a scanning electron microscope (Zeiss EVO MA 10, Germany). The diameters of CNF were then measured using an image analyzer (ImageJ, NIH, USA) [21].

### Transmission electron microscopy (TEM)

For TEM analyses, the cellulose and CNF (0.01 wt%) were deposited on a copper grid and observed under a transmission electron microscope (TECNAI G2 T20 TWIN, US) operated at 100 kV. The width of the fibers was measured for 200 individual nanofibrils using an image analyzer (ImageJ, NIH, USA).

### Brunauer–Emmett–Teller (BET) analysis and pore size distribution

The Brunauer–Emmett–Teller specific surface area (BET) was determined by nitrogen physisorption using an Autosorb iQ (Quantachrome Instrument, USA). About 0.1–0.2 g of the freeze-dried cellulose nanofibre sample was degassed at 115°C for 18 h prior to the analysis, followed by N_2_ adsorption at 196°C. A BET analysis was carried out for a relative vapor pressure (P/P_0_) of 0–1 at −196°C. The average pore size of the CNF was estimated from the nitrogen desorption isotherm, according to the analysis of Barrett–Joyner–Halender [22].

### Measurement of transmittance of CNF through UV−visible spectroscopy

The CNF dispersions were prepared at 0.05 wt% (w/v) in milli-Q, and the suspension was then analyzed using a UV Visible Spectrophotometer (Shimadzu, UV-2450, Japan) in the spectra of 800 − 400 nm. The polystyrene cuvettes used were degassed through a sonicator for 5 min before the analysis [8].

### Carboxyl group density of TEMPO oxidized cellulose

The conductivity titration technique was used to determine the carboxylate content of the TEMPO oxidized CNF. The dried CNF sample (0.3 g) was added in 55 mL of water and 5 mL of 0.01 M NaCl and the mixture was stirred to prepare a homogenous mixture. Then, 0.1 M HCl was added to protonate all the carboxyl groups till the pH value reached at 2.0. Then, the solution was titrated against 0.04 M NaOH solution. The amount of carboxylate content of the sample was determined using Eq. (2) [7]:

(2) $$Carboxyl\;group\;density\left(mmol/g\;\right)\;=\;NaOH\;concentration\;\times \;Volume\;of\;NaOH\;used\;÷\;mass\;of\;CNF$$

### Contact angle of the CNF sheet

The water contact angle (WCA) measurements were performed with the help of automatic multi liquid dispenser of Theta flow tensiometer (Biolin Scientific). Deionized water (10 μL) was deposited on a sheet, and data was taken at 0.33 sec interval till 25 sec. The contact angle was calculated by Young’s equation. The average of the contact angles was calculated to determine the wettability of the CNF sheet [23].

### Statistical analysis

All the experiments were performed in triplicates to minimize handling errors. The error bars represent the standard deviation among the triplet of samples.

## Results and discussion

The agro-waste rice straw was used as a raw material in the study to extract cellulose and its nanoform *viz*. cellulose nanofibres that were further characterized for their morphological, functional and surface charge variation. The yield percentage of the cellulose nanofibers was calculated using Eq. (1) which was around 70%. The yield of the process was estimated in triplicates.

### Functional characterization of cellulose and cellulose nanofibres

**Figure 3.**
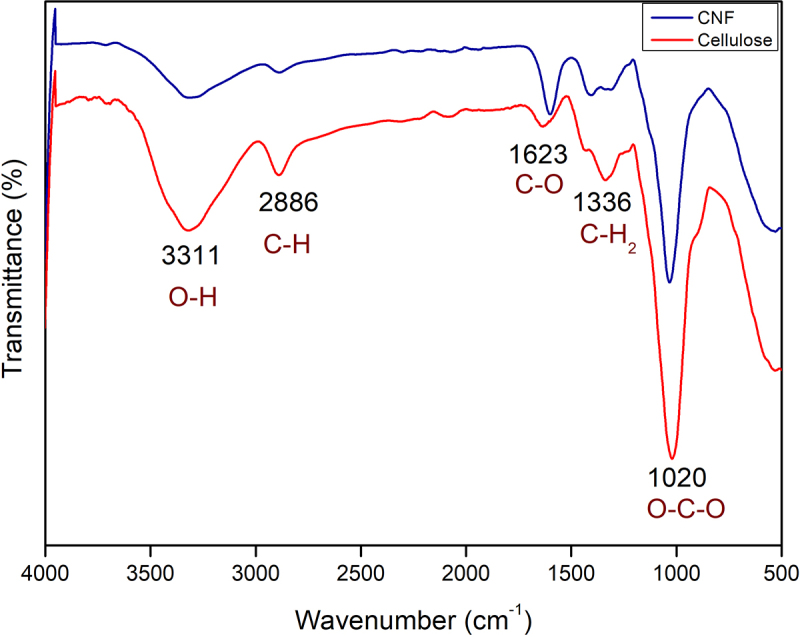
FTIR spectra of cellulose and cellulose nanofibers (CNFs).

The infrared spectra of extracted cellulose and CNF were taken through FTIR spectroscopy to determine the chemical structure. No structural change was observed in the two spectra, as shown in Figure 3. The peak corresponding to the -OH vibration in the cellulose and CNF appeared at 3311 cm^−1^ [24]. The peak at 2886 cm^−1^ corresponds to the tensile vibration of C-H bonds in cellulose and CNF groups [25]. In this study, the peaks around 1623 and 1336 cm^−1^ wavenumber assigned to the symmetric bending of CH_2_ and the bending vibrations of the C-O groups of the rings in polysaccharides, respectively, were also observed. The sharp band at 1623 cm^−1^ assigned to the stretching vibration of C-O which was seen in CNF because of the TEMPO treatment the C6 carbon was oxidized to COO^−^. Furthermore, the peak at 1020 cm^−1^ was attributed to O-C-O stretching rocking vibrations of the pyranose ring skeleton and assigned to the carbohydrate rings of the cellulose skeleton. The characteristic bands for stretching vibrations of xylene and syringyl ring found in lignin and hemicelluloses were assigned to wavenumber 1243 cm^−1^ [26] which was absent in both the samples because of the bleaching and alkali treatment. Similar peaks were also observed in many reports that showed the prominent peak at 1020 cm^−1^of glucopyranose, which remains stable even after pre-treatment [27,28].

### Zeta potential of cellulose and cellulose nanofibres

The zeta potential of cellulose and cellulose nanofibers was measured using a zeta sizer. Owing to the presence of hydroxyl group on the cellulose, the surface of the cellulose was negatively charged (−19.0 mV). The CNFs were found to have a significantly higher negative surface charge (−42.0 mV) than the cellulose, which was attributed to the inclusion of the -COO group at the C6 carbon position of the cellulose after TEMPO treatment (Figure S2). The zeta potential results from this study were consistent with another study where the CNF was passed through a homogenizer with 120 passes, showing a surface charge of −37.52 mV, and with 50 passes, the surface charge was −40.98 mV [29]. Due to the electrostatic repulsion, forces between the fibers help to segregate the fibrils and make them stable under colloidal dispersion [14]. The high carboxyl content facilitates the ionic binding of fertilizer for later studies.

### Morphological characterization of cellulose and cellulose nanofibres

**Figure 4.**
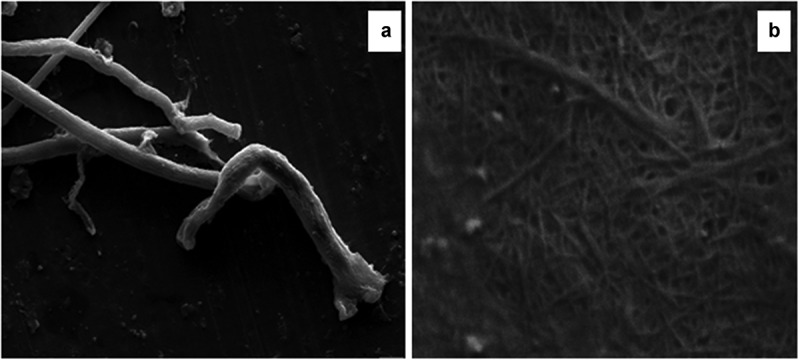
Scanning electron micrographs of cellulose and cellulose nanofibers (CNFs) at 20 kV and magnification of 50 kX.

To understand the impact of TEMPO oxidation on the morphology of cellulose, scanning electron microscopy (SEM) analysis was performed for cellulose and cellulose nanofiber samples. The scanning electron micrograph of cellulose represents elongated fibers with a micrometric diameter (4.34 ± 2 µm). As seen in Figure 4, there was a significant morphological variation in terms of fiber shapes and thickness after the conversion of cellulose fibers to nanofibers mediated by TEMPO treatment. The diameter of cellulose nanofibres was 17 ± 4 nm. It was observed that the fiber structure varied in diameters after the oxidation process. Reports showed that the longer oxidation time (120 min) impacted the structural integrity of the fibers, due to which either the fibers unfolded or curled, the fibers appeared flattened [14]. Furthermore, there is a correlation between the surface roughness and carboxyl content of fibers. A report stated that a decrease in the surface roughness of fiber was seen with a higher carboxylic content [20]. Recent research has demonstrated that self-fibrillation or auto-liberation of nanofibrils during sequential TEMPO/periodate oxidation is conceivable as long as the surface charge is sufficient [30,31].

**Figure 5.**
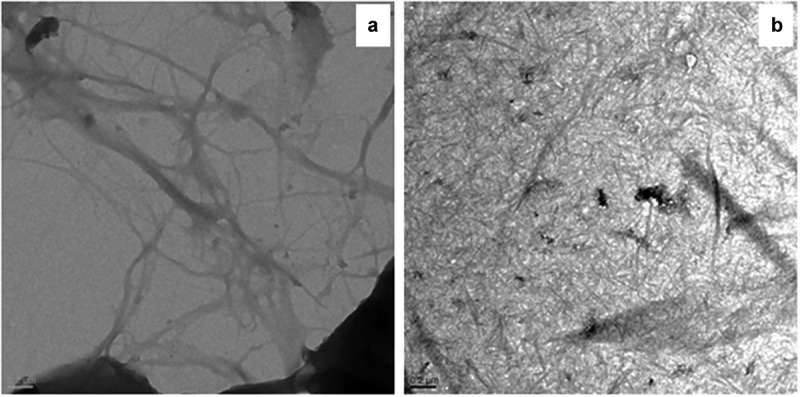
Transmission electron micrographs of cellulose and cellulose nanofibers (CNFs) at the scale bar of 200 nm.

Transmission electron micrographs of cellulose showed the interlinked and large fibers with a micrometric diameter (1.6 µm), whereas the CNF was found to be well-arranged thread like network with the diameter of 15–20 nm, confirming the effective oxidation process of cellulose fibers (Figure 5). Other study by Islam et al. on nanocellulose extracted from rice straw exhibited average diameter of 10–50 nm [31]. Thus, nanofibers generated in the present study had lower diameter than CNF already report in the literature. It is clear that processes of soda pulping, bleaching efficiently eliminated the non-cellulosic components, hence lowering the fiber dimension.

### BET and porosity characteristics of CNF

**Figure 6.**
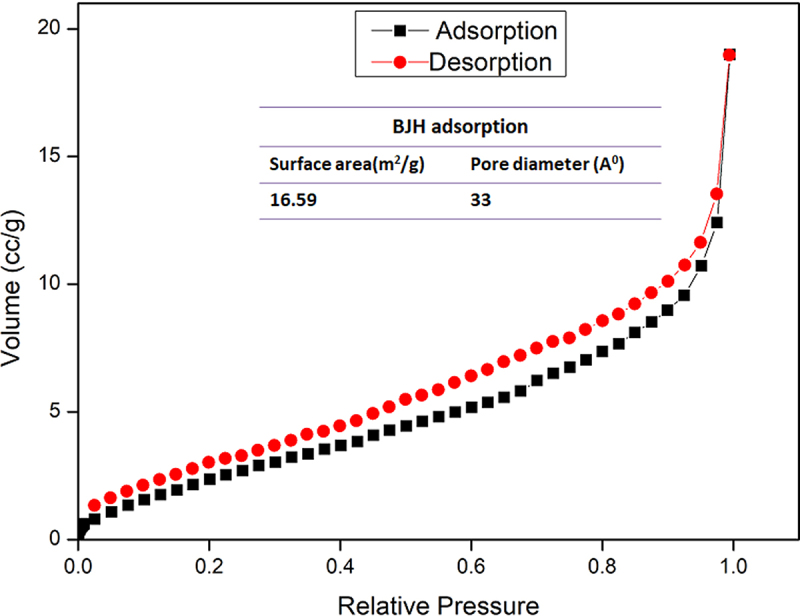
Gas adsorption-desorption isotherm of CNF.

The surface area and pore size distribution of the CNFs were examined using the nitrogen adsorption-desorption isotherms and Barrett–Joyner–Halenda (BJH) adsorption desorption isotherms from BET (Brunauer–Emmett–Teller) analysis. According to Figure 6, the CNFs displayed type IV hysteresis type 3, which is typical for raw cellulose and involves mesoporous adsorbents. Emmanuel et al. observed a similar type of hysteresis loop when they synthesized CNFs from absorbent cotton with pore diameters of 44.89 °A and surface areas of 0.38 m^2^/g [32].

The BET surface area for CNF was found to be 16.59 m^2^/g. The BJH pore size distribution of the CNF from adsorption isotherms was very sharp, and the pore diameter of the CNF was recorded to be 33 °A. However, it was anticipated that CNFs contain a stiff, nearly unbroken hydrogen bond that may have an impact on pore size [33]. Further, the porosity, pore widths, and density are all influenced by the concentration of the sample. As concentration rises, the structure becomes denser and more compact, which results in less porosity and smaller pore diameters [8]. Surface area and pore size determination is a complex venture that varies the theoretical values among different parent materials [34].

### Transmittance and carboxyl content of CNF suspension

**Figure 7.**
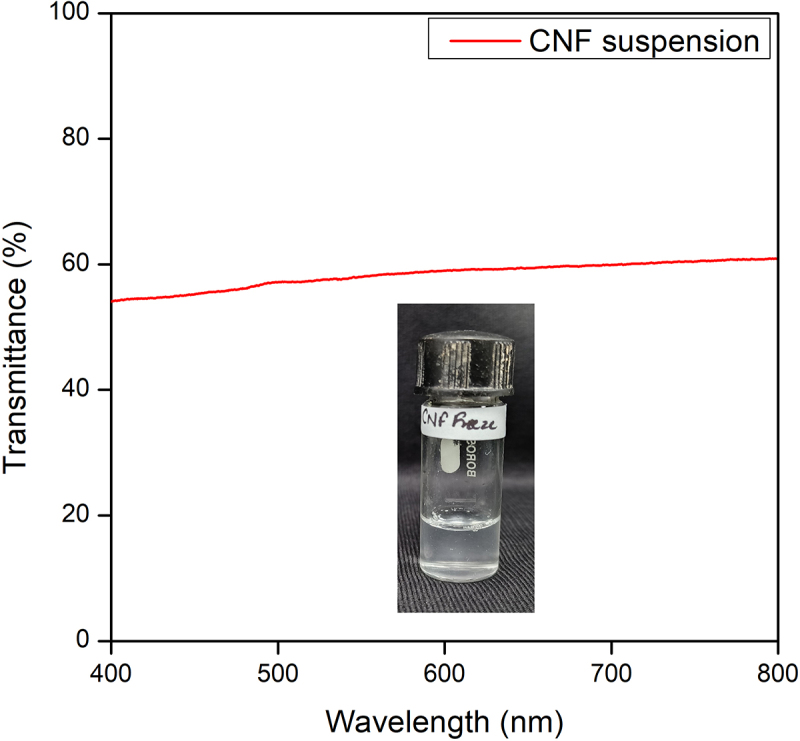
Light transmission spectra of CNF suspension.

The transmittance for the CNF dispersions was recorded from 800 to 400 nm by UV Vis spectra to study the dispersion turbidity, which is proportional to the particle and the degree of fibrillation (Figure 7). In this study, CNF showed a transmittance of more than 50%. Larger particles, such as defibrillated fragments or agglomerates, scatter incident light more effectively, lowering transmittance as reported by Parit et al. and Zhang et al. [35,36]. Whereas, in the case of TEMPO-oxidized CNF, the fiber diameters are only a few nanometers, so the small fibrils did not effectively scatter incident light, which contributed to the high light transmittance value [37,38]. Another report showed that CNF passed through different passes of the homogenizer showed 50–79% transmittance through UV visible spectroscopy. Hence, the higher the transmittance percentage, the finer the CNF [14]. Based on the NaOH consumed in the plateau regions, the carboxyl group density was calculated to be 2.66 mmol C6 carboxyl per g of cellulose nanofibres. This shows that the electrostatic repulsion between the CNFs is strong enough to keep them widely scattered and make them available for ionic interaction with the nitrogen-based fertilizer.

### Wettability of the CNF sheet

**Figure 8.**
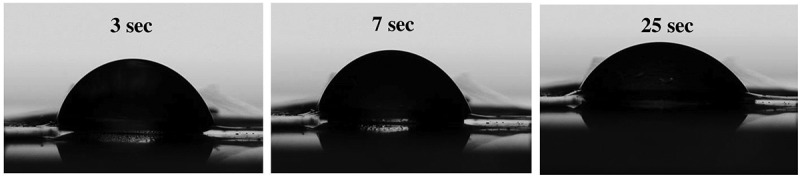
FTIR spectrum of ammonium chloride and loaded samples (1:1, 1:2, and 2:1).

The contact angle images of the CNF sheet were captured at 3, 7 and 25 sec and were shown in Figure 8. The water contact angle of CNF sheet after 25 sec was 50.27 ± 3.83°. The hydroxyl group of cellulose was oxidized to carboxyl group during TEMPO oxidation. The carboxyl group tends to enhance the hydrophilicity because the functional group induces ionic bond interaction [39]. There are two major factors that influence the water contact angle; one is the lignin content that contributes to the hydrophobic nature so the initial contact angle of the wood source was higher due to the presence of lignin. The lignin content tends to decrease the surface energy of the fibrils. Second major parameter is surface roughness of the CNF sheet, which led to the development of air pockets between rough surface and the droplet [40,41].

### Loading and release of ammonium ion loaded on to CNF

**Figure 9.**
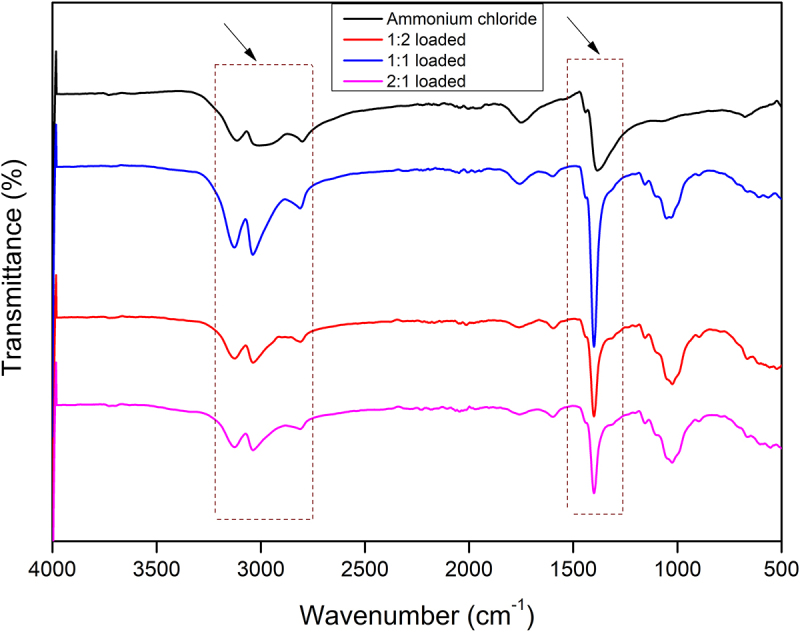
Release behavior of ammonia from loaded and control CNF.

Owing to the negative charge on the surface of CNF, it was expected that positively charged ammonium ion will be readily loaded onto the CNF via an electrostatic/ionic interaction. Ammonium chloride was hence loaded onto CNF at three different ratios (1:1, 1:2, and 2:1 (CNF: NH_4_Cl) w/w). The loading of ammonium ions onto CNF was confirmed through FTIR spectroscopy and Zetasizer. The FTIR spectra of all three loaded CNF samples (1:1, 1:2, and 2:1) indicated peaks in the regions 3200–2800 cm^−1^ and 1459 cm^−1^ which confirmed the presence of ammonium ions in the loaded CNF samples. The maximum peak intensity of ammonium ions was found in 1:1 loaded samples where the concentration of ammonium chloride was doubled (Figure 9). The surface charge estimation via Zetasizer indicated the reduction of negative charge (−42.0 mV) from the surface of the loaded CNF samples due to the loading of ammonium ions. In the loaded sample, the surface charge was reduced to −12.8 mV, −25.0 mV, and −28.0 mV, respectively, in CNF loaded with ammonium chloride at a 1:1, 1:2, and 2:1 ratio. The maximum loading was seen in the 1:1 loaded samples. The amount of ammonium loaded onto the CNF samples was determined using Kjeldahl method. The loading percentage of ammonium ions was significantly higher in 1:1 loaded samples, which were around 55%, whereas in 1:2 and 2:1 the amount was around 32% and 30%.

#### Release kinetics

**Figure 10.**
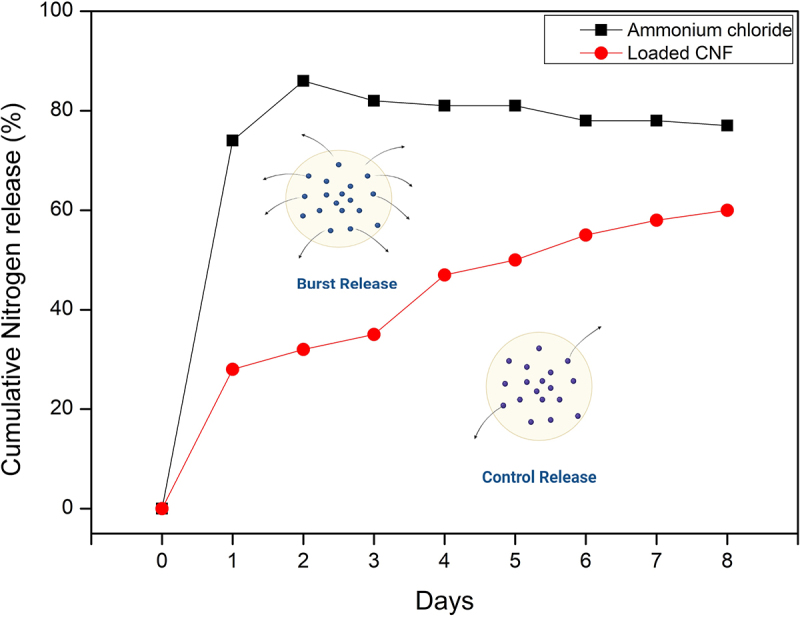
Release behavior of Ammonia from loaded CNF (1:1) and control (ammonium chloride solution)

A simple diffusion experiment was carried out to analyze the rate of nutrient release in the water from loaded and control CNF. The nutrient release plot as depicted in Figure 10 clearly depicts the controlled release behavior of the CNF loaded sample compared to the control. The controlled release mechanism of CNF-based fertilizer involves the dissociation of ammonium ions that are attached to the negatively charged CNF through ionic interaction and the process follows the basic principle where diffusion occurs because the concentration of ammonium ions attached to the carrier (CNF) is higher in the dialysis bag than in the surrounding environment. The release of ammonium ions was fastest in cases of positive control, where the ammonium chloride solution was added directly to the dialysis bag. However, in the case of loaded CNF, the rate of ammonium ions was arrested, with a release of 58% in 8 days. The N-release profile followed an analogous parabolic diffusion process [42]. Peng et al. reported a cumulative nitrogen release of around 47.3% within 7 days from a biochar-based slow release nitrogen fertilizer [43,44].

Therefore, it is necessary to discuss the controlled-release mechanism of CNF-based fertilizer. The controlled release mechanism of coated fertilizers on CNF may follow the pattern of parabolic release through the linear pattern to sigmoidal release as the normal pattern of release of nutrients and plant uptake [45]. Water tends to penetrate the coating, and the vapor condenses within the solid core, creating internal pressure that the CNF core resists, and the fertilizer is released through diffusion driven by a concentration gradient across the coating [45]. Hence, the study showed that the cellulose nanofibers derived from agro-waste have higher carboxyl content with −42.0 mV surface charge that helps in the ionic interaction with the agrochemical so, can be utilized as a carrier system for delivering various agrochemicals in a controlled release pattern.

## Conclusions

Rice straw-based cellulose nanofibers were used as a carrier system for the controlled delivery of fertilizers to plants in this study. As a result, the loading and release studies of CNF loaded with ammonium chloride revealed a controlled pattern with of 58% release in 8 days, meeting expectations as a viable controlled-release fertilizer (CRF) system that may be effective in reducing losses through leaching, whereas the ammonium chloride control revealed a release rate of more than 80% after 8 days.

## Supplementary Material

Supplemental Material

## Data Availability

Data openly available in a public repository that issues datasets with DOIs.
