# Supplementary material for: Controlled release fertilizer delivery system derived from rice straw cellulose nanofibres: a circular economy based solution for sustainable development
Source: Bioengineered. 2023 Aug 7;14(1):2242124. doi: 10.1080/21655979.2023.2242124 (PMC10408692; doi:10.1080/21655979.2023.2242124)
Supplement: Supplemental Material [file KBIE_A_2242124_SM0073.docx]

Supplementary data

**Controlled release fertilizer delivery system derived from rice straw cellulose nanofibres: A circular economy based solution for sustainable development**

Neha Sharma^1, 2^, Benjamin James Allardyce^2^, Rangam Rajkhowa^2^, Ruchi Agrawal^1*^

^1^TERI Deakin Nanobiotechnology Centre, TERI Gram, Gwal Pahari, Gurugram, India

^2^Deakin University, Institute for Frontier Materials, Geelong, Australia

^*^Corresponding author.

E-mail: ruchi.agrawal@teri.res.in

**Experiment 1**: Estimation of quantity of ammonium ion from the loaded samples through nesseler’s reagent.

The calibration curve covered the concentration range of 0 to 0.5 mg/ ml and the R^2^ value was 0.99. Nessler’s reagent is composed of Potassium tetraiodomercurate which in the presence of ammonia generate brown fumes and brown precipitate and showed absorbance at 420 nm.

Figure S1. Calibration curve of ammonium chloride (mg/ml)

The slope equation is used to determine the concentration of ammonia in the loaded samples (in mg). The equation is:

$Y=mx\pm c$ (1)

Where, the y is the absorbance at 420 nm, m is 6.5257 and c is 0.0686. The x (concentration of ammonia) is determined through the equation below derived from equation 1.

$x=Y\pm c\div m$ (2)

**Experiment 2**: Determination of surface charge of cellulose and cellulose nanofibres through zetasizer

**
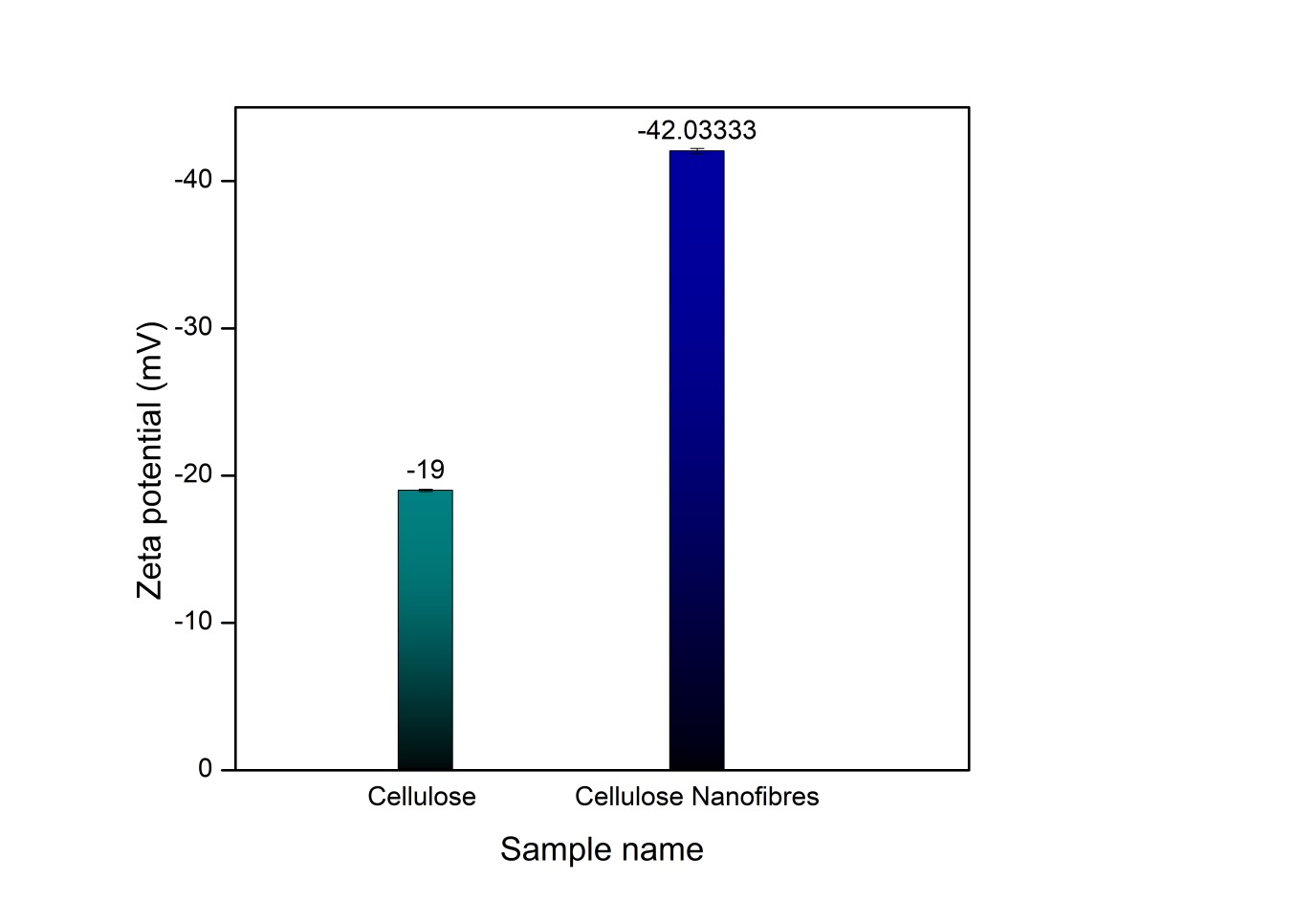
**

Figure S2. Zeta potential of cellulose and cellulose nanofibres

**Experiment 3:** Optimization of TEMPO oxidation through CNF

The process was optimized by changing the concentrations of sodium hypochlorite and dispersibility was monitored after 24 hours. Followed by, morphological analysis of the samples through SEM which reflect that mentioned protocol (around 500mg of extracted cellulose was added in 37.5mL of water and stirred for 5min using magnetic stirrer (Tarson digital spinot). Around 6.25mg of TEMPO and 62.5mg of sodium bromide were then added under continuous stirring for another 5min. Further, 5mM solution of sodium hypochlorite was added drop-wise to initiate the oxidation process) appeared suitable for nanofibre production from the cellulose.


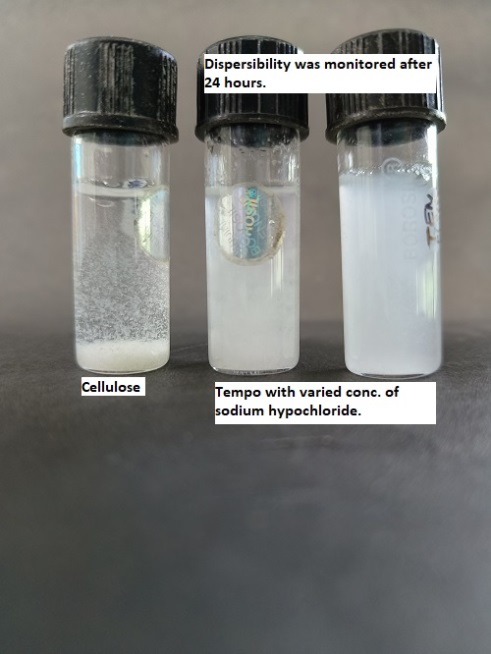

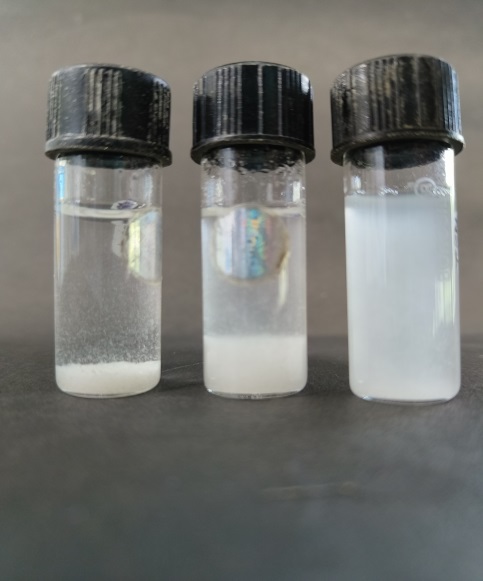


After 24 hours

Figure S3. Dispersion of TEMPO treated cellulose

Figure reflects the dispersion is less in cellulose and second bottle. The scanning micrographs further confirm the nano-meteric diameter of the fibres.


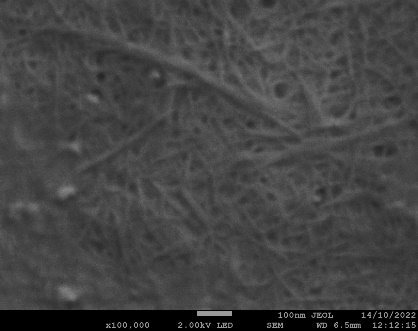


Cellulose fibres derived from rice straw (Parent fibres)

TEMPO+ 2mM conc. of sodium hypochlorite

TEMPO+ 5mM conc. of sodium hypochlorite


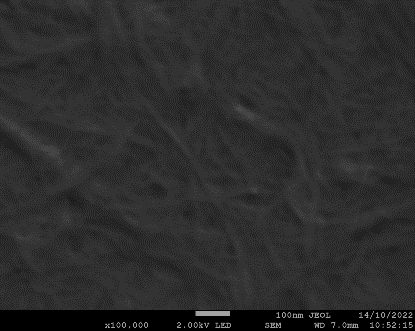

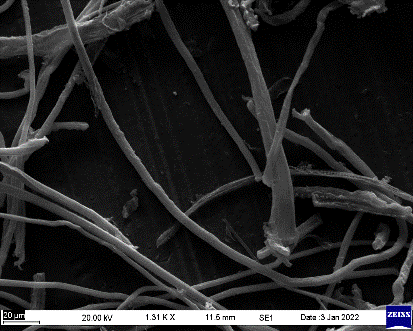


Figure S4. Scanning electron micrograph of cellulose and TEMPO treated samples


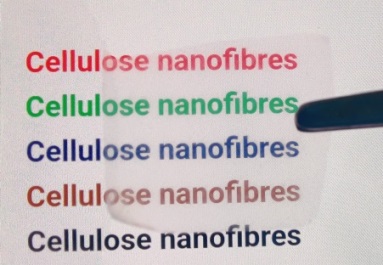
Similarly, variation of TEMPO and other salts were made to achieve the optimum protocol and to get the transparent sheet of CNF that can be dispersed easily and can facilitate the binding of fertilizers.

Figure S5. TEMPO prepared CNF sheet with 5mM conc. Of sodium hypochlorite
